# A centrosome clustering protein, KIFC1, predicts aggressive disease course in serous ovarian adenocarcinomas

**DOI:** 10.1186/s13048-016-0224-0

**Published:** 2016-03-18

**Authors:** Karuna Mittal, Da Hoon Choi, Sergey Klimov, Shrikant Pawar, Ramneet Kaur, Anirban K. Mitra, Meenakshi V. Gupta, Ralph Sams, Guilherme Cantuaria, Padmashree C. G. Rida, Ritu Aneja

**Affiliations:** 1 Department of Biology, Georgia State University, Atlanta, GA 30303 USA; 2 Department of Mathematics, Science and Bioinformatics, Mercer University, Atlanta, GA USA; 3 Department of Medical and Molecular Genetics, Medical Sciences Program, Indiana University School of Medicine, Bloomington, IN USA; 4 Department of Pathology, West Georgia Hospital, Lagrange, GA USA; 5 Department of Pathology, Northside Hospital, Atlanta, GA USA; 6 Department of Gynecologic Oncology, Northside Hospital Cancer Institute, Atlanta, GA USA; 7 Novazoi Theranostics, Plano, TX USA

**Keywords:** Centrosome amplification, Centrosome clustering, KIFC1, Serous ovarian adenocarcinoma

## Abstract

**Background:**

Amplified centrosomes are widely recognized as a hallmark of cancer. Although supernumerary centrosomes would be expected to compromise cell viability by yielding multipolar spindles that results in death-inducing aneuploidy, cancer cells suppress multipolarity by clustering their extra centrosomes. Thus, cancer cells, with the aid of clustering mechanisms, maintain pseudobipolar spindle phenotypes that are associated with low-grade aneuploidy, an edge to their survival. KIFC1, a nonessential minus end-directed motor of the kinesin-14 family, is a centrosome clustering molecule, essential for viability of extra centrosome-bearing cancer cells. Given that ovarian cancers robustly display amplified centrosomes, we examined the overexpression of KIFC1 in human ovarian tumors.

**Results:**

We found that in clinical epithelial ovarian cancer (EOC) samples, an expression level of KIFC1 was significantly higher when compared to normal tissues. KIFC1 expression also increased with tumor grade. Our *In silico* analyses showed that higher KIFC1 expression was associated with poor overall survival (OS) in serous ovarian adenocarcinoma (SOC) patients suggesting that an aggressive disease course in ovarian adenocarcinoma patients can be attributed to high KIFC1 levels. Also, gene expression levels of KIFC1 in high-grade serous ovarian carcinoma (HGSOC) highly correlated with expression of genes driving centrosome amplification (CA), as examined in publically-available databases. The pathway analysis results indicated that the genes overexpressed in KIFC1 high group were associated with processes like regulation of the cell cycle and cell proliferation. In addition, when we performed gene set enrichment analysis (GSEA) for identifying the gene ontologies associated to KIFC1 high group, we found that the first 100 genes enriched in KIFC1 high group were from centrosome components, mitotic cell cycle, and microtubule-based processes. Results from in vitro experiments on well-established in vitro models of HGSOC (OVSAHO, KURAMOCHI), OVCAR3 and SKOV3) revealed that they display robust centrosome amplification and expression levels of KIFC1 was directly associated (inversely correlated) to the status of multipolar mitosis. This association of KIFC1 and centrosome amplification with HGSOC might be able to explain the increased aggressiveness in this disease.

**Conclusion:**

These findings compellingly underscore that KIFC1 can be a biomarker that predicts an aggressive disease course in ovarian adenocarcinomas.

**Electronic supplementary material:**

The online version of this article (doi:10.1186/s13048-016-0224-0) contains supplementary material, which is available to authorized users.

## Background

Ovarian cancer is the sixth most common cancer affecting women worldwide and is the fifth leading cause of deaths related to gynecological malignancies with less than 40 % overall cure rate [1]. The overall mortality of ovarian cancer has remained largely unchanged over the past decades even though there is a great advancement in surgical and therapeutic approaches [2]. The standard treatment for ovarian cancer patients is debulking surgery followed by a platinum- based chemotherapy (cisplatin and carboplatin) [3, 4]. One of the primary causes of the high mortality and poor survival in ovarian cancer is the diagnosis at late stages [5]. Despite years of extensive research, there is still a dearth of reliable biomarkers for early detection, prognosis, and predicting disease aggressiveness. Since ovarian cancer is a heterogeneous disease with different histopathological features and clinical behavior, a better understanding of molecular subtypes and search for clinically-facile prognostic factors that can aid in histological subtyping is imperative. Greater than 90 % of malignant ovarian tumors are epithelial ovarian carcinomas (EOC) comprising of various subtypes namely serous, endometrioid, clear cell, transitional cell, squamous cell and mucinous carcinomas [6, 7]. About 70–80 % of all cases are serous ovarian cancer (SOC) among which high-grade serous ovarian cancer (HGSOC) is the most prevalent [8]. Intriguingly, HGSOC shares similar genomic features with triple negative breast cancer (TNBC) as per reports from Cancer Genome Atlas (TCGA) Network analysis; in particular, the deregulated pathways characterizing HGSOC are very similar to those in TNBC [9]. Several independent studies have indicated that HGSOC is associated with very high genomic instability and chromosomal aberrations including intrachromosomal breaks and aneuploidy, which incidentally, also typify and drive intratumoral heterogeneity in TNBC [10, 11].

Specifically, the most common mutations present in both kinds of tumors (HGSOC and TNBC) are of p53 and BRCA1/2. It is well established that BRCA1 and BRCA2 tumor suppressor genes directly preserve genomic stability by regulating DNA repair, p53-mediated cell cycle checkpoint control as well as centrosome duplication cycle [12–14]. These findings establish the causative link between BRCA1 and BRCA2 mutations and extensive chromosomal instability found in HGSOC patients. Furthermore, HGSOC tumors frequently overexpress cyclin E and Aurora-A, resulting in aberrant activation of the centrosome duplication cycle that induces centrosome amplification (CA), and eventually genetic instability fueling ovarian cancer progression [15–17]. CA results in numerous and voluminous centrosomes [18]. Subjectively, the presence of supernumerary centrosomes sets the stage for the formation of multipolar spindles that may succumb to a mitotic catastrophe. However, cancer cells avoid this calamitous fate by clustering their extra centrosomes at the two spindle poles, which allows them to evade cell death but ultimately engenders low-grade aneuploidy and genetic instability [19–21].

KIFC1, a nonessential kinesin motor protein, also known as HSET, plays a critical role in clustering of extra centrosomes in cancer cells. Recently several studies have shown that knockdown of KIFC1 in cancer cell lines containing supernumerary centrosomes causes the excess centrosomes to be scattered by pole-separating forces that induce spindle multipolarity and cell death. However, KIFC1 is not required for bipolar spindle assembly in healthy somatic cells [22, 23]. We recently demonstrated that EOC clinical samples harbor extra centrosomes and display high levels of centrosome clustering in interphase as well as mitosis. In addition, the study highlighted that the gene expression levels of KIFC1 are higher in EOC when compared to normal ovarian tissues *in silico* and is associated with worse prognosis and survival [24]. To further understand and validate results of our previous study, we herein evaluated KIFC1 expression in clinical samples of ovarian cancer by utilizing immunohistochemical staining. Our results indicated higher KIFC1 expression in EOC tumor samples when compared to normal tissues. Furthermore, KIFC1 expression levels in EOC increased with an increase in tumor grade. To understand better the association of KIFC1 with CA, we examined correlations between expression levels of KIFC1 and genes driving CA. Intriguingly, higher gene expression levels of KIFC1 was significantly correlated to expression of CA-driving genes. When GSEA was performed for the genes enriched in KIFC1-high group, they were also found to be related to centrosome components and microtubule-based processes. We further validated the correlation by doing quantitative analysis of CA and extent of clustering in cell lines derived from SOC patients. Our results indicated that KIFC1 was highly expressed in these in vitro models of SOC and was also associated to levels of centrosome clustering (mitotic), enabling cells to bypass mitotic catastrophe.

Taken together our findings underscore that KIFC1 is a potential prognostic biomarker in ovarian adenocarcinomas wherein expression levels of KIFC1 may predict the course of disease aggressiveness. Work is underway in our laboratory to pin point molecular mechanism to explain the association of KIFC1 and CA with ovarian cancer aggressiveness and poor patient outcomes.

## Results

### KIFC1 is overexpressed in Epithelial Ovarian adenocarcinoma (EOC) clinical samples

**Fig. 1:**
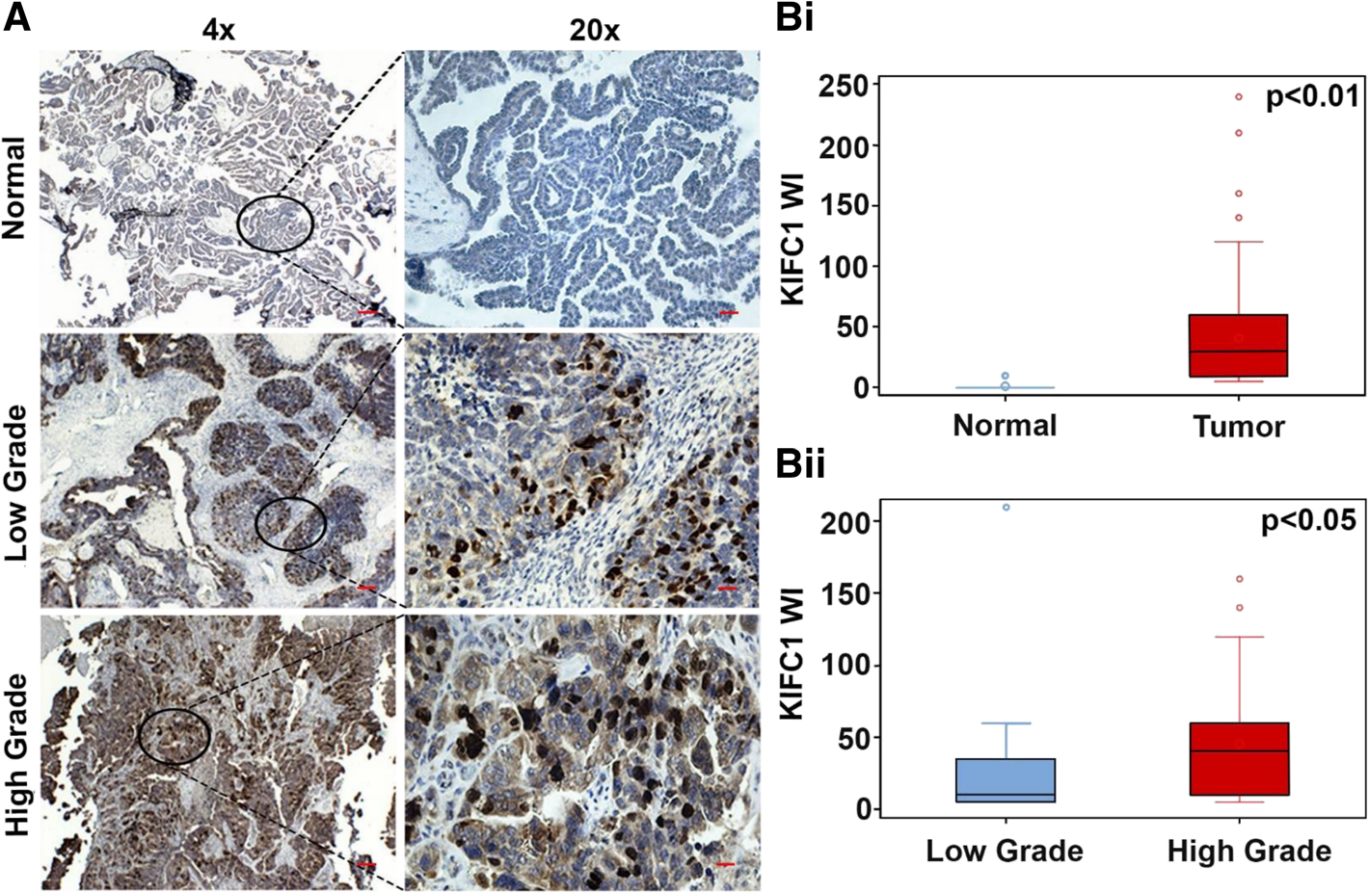
High grade epithelial ovarian carcinomas exhibit higher expression of KIFC1 than low-grade adenocarcinomas and uninvolved, adjacent normal tissues. **A** Low magnification (4x) and their corresponding higher magnification (20x) images depicting KIFC1 expression in normal, low-grade and high-grade EOC tissues. The tissues were stained for KIFC1 (brown) and nuclei (blue). Scale bar (red) 20 μm. **Bi** Box-whisker plot depicting the weighted index (WI) of KIFC1 expression in normal and tumor tissue. **Bii** Box-whisker plot representing the WI for KIFC1 expression in low and high-grade EOC samples

We first examined whether KIFC1 is upregulated in human ovarian cancers by analyzing KIFC1 overexpression in EOC clinical samples. To this end, we immunostained paraffin-embedded formalin-fixed tissue microarrays of EOC (*n* = 120) and normal ovarian epithelial tissue (*n* = 13) for KIFC1. The staining intensity was scored as 0 = none, 1 = low, 2 = moderate, or 3 = high, and percentage of positive cells (i.e., with 1+ staining intensity) from randomly selected fields (~500 cells) was determined [18]. The product of the staining intensity and the percent of positive cells constituted the Weighted Index (WI). Descriptive statistics regarding patient and clinicopathological characteristics is given in Tables 1, 2 and 3. In consonance with our previously published study [24], our immunohistochemical analysis showed overexpression of KIFC1 in EOC tissues with negligible expression in normal ovarian epithelial tissue (Fig. 1A). We found that the number of positively-stained nuclei per field in high-grade ovarian cancers (Fig. 1A) was significantly higher compared to low-grade ones. We then compared the nuclear KIFC1 WI values for normal and tumor samples and also across grades for tumor samples. Interestingly, we observed that nuclear KIFC1 WI was significantly higher in EOC tissues when compared to normal tissues (*p* < 0.01). Also, the nuclear KIFC1 WI increased with increasing tumor grade (Figure 1Bii) (*p* < 0.05). Among subtypes, we noticed that the number of positively-stained nuclei per field in high-grade serous ovarian cancers (Additional file 1: Figure S1A) was significantly higher compared to low-grade serous ovarian cancers (*p* < 0.05). Collectively, these observations indicate robust KIFC1 overexpression in human ovarian adenocarcinoma and strong association of KIFC1 expression levels with clinical progression of the disease. These data suggest that KIFC1 might play an active role in driving the progression of tumors into more malignant and aggressive forms.

**Table 1:** Descriptive statistics for patient and clinicopathologic characteristics in the analysis of KIFC1 levels in tumors and matched normal tissue SD = standard deviation

Variable	Level	Number	Percentage
Age	20–40	23	19.2
	41–60	81	67.5
	61<	16	13.3
Grade	1	32	26.7
	2	36	30
	3	46	38.3
	Unknown	6	5
Stage	I	69	57.5
	II	31	25.8
	III	12	10
	IV	3	2.5
	Unknown	5	4.2
Primary Tumor (T)	T1	72	60
	T2	31	25.8
	T3	12	10
	Unknown	5	4.2
Regional Lymph Nodes (*N*)	N0	103	85.8
	N1	12	10
	Unknown	5	4.2
Distant metastasis	Yes	3	2.5
	No	112	93.3
	Unknown	5	4.2
Tissue type	Malignant	115	95.8
	Metastasis	5	4.2

**Table 2:** Description of the subtypes of epithelial ovarian cancer for clinical samples included in the anlaysis of KIFC1 levels

Variable	Level	Number	Percentage
Pathological diagnosis	Adenocarcinoma	2	1.7
	Serous Adenocarcinoma	6	5
	Serous Papillary Adenocarcinoma	3	2.5
	Endometrioid Adenocarcinoma	10	8.3
	Metastatic Adenocarcinoma	4	3.3
	Metastatic Mucinous Adenocarcinoma	1	0.8
	Mucinous Adenocarcinoma	11	9.2
	Clear Cell Carcinoma	4	3.3
	Serous Papillary Carcinoma	32	26.7
	Serous Papillary Cystadenocarcinoma	47	39.2

**Table 3:** Descriptive statistics and clinicopathologic characteristics for patients included in *in silico* analysis of KIFC1 expression and overall survival

Variable	Level	Number	Percentage
Age (Range)	20–29	1	0.5
	30–39	4	1.9
	40–49	23	11.1
	50–59	84	40.4
	60–69	54	26
	70–79	40	19.2
	80–89	1	0.5
	Unknown	1	0.5
Cancer site	Primary	154	74
	Metastasis	50	24
	Unknown	4	1.9
FIGO Stage	I	9	4.3
	II	9	4.3
	III	126	60.6
	IV	10	4.8
	Unknown	54	26
Grade	1	6	2.9
	2	80	38.5
	3	120	57.7
	Unknown	2	1
Survival Status	Alive	109	52.4
	Dead	98	47.1
	Unknown	1	0.5
Recurrence	Recurrence	154	74
	No Recurrence	53	25.5
	Unknown	1	0.5

### Enhanced KIFC1 gene expression is associated with poor survival in HGSOC patients

**Fig. 2:**
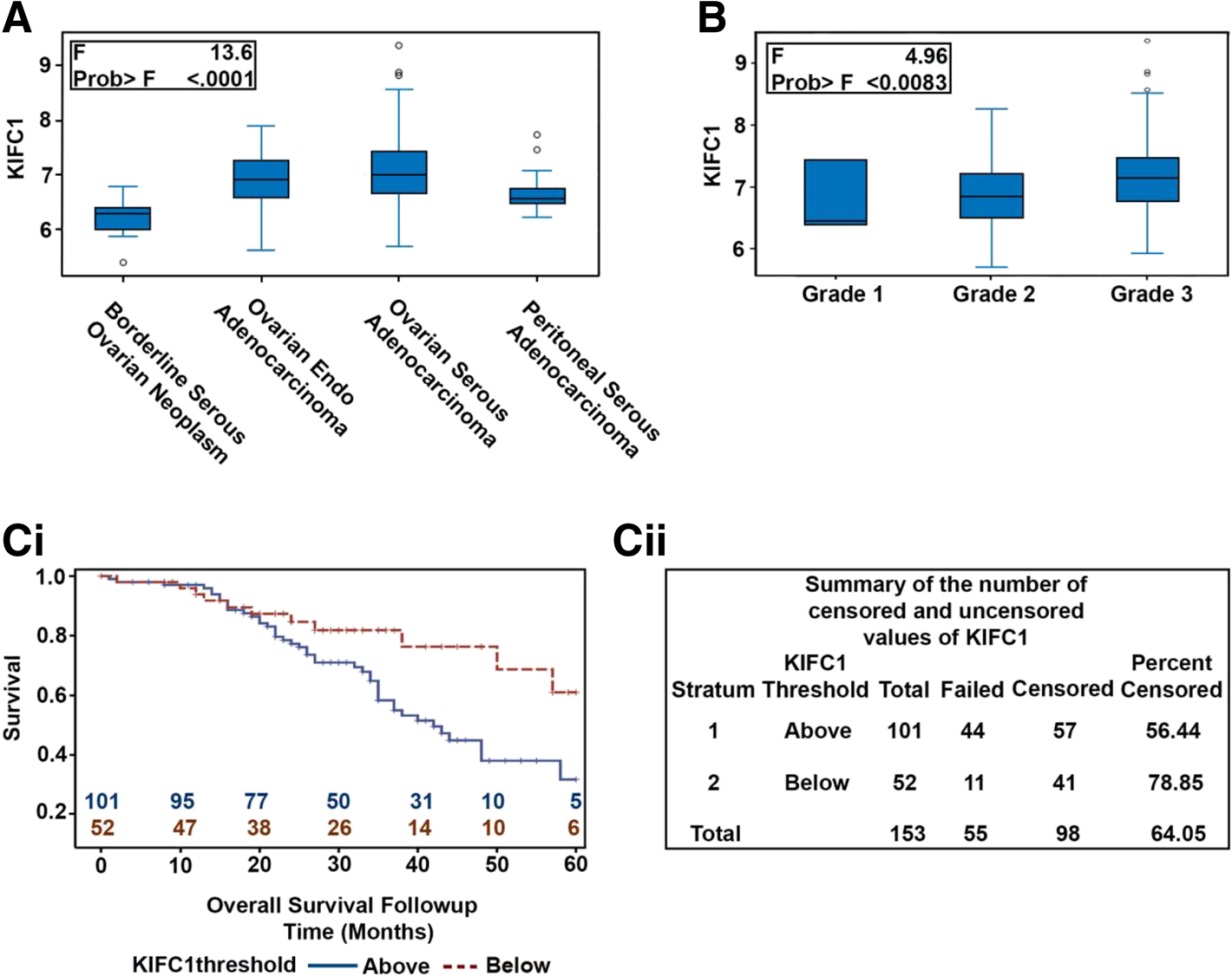
KIFC1 is highly expressed in High grade serous ovarian adenocarcinoma and is associated with poor overall survival. **A** Box-whisker graphs depicting the expression levels of KIFC1 among different subtypes of ovarian cancer. **B** Box- whisker graphs depicting the expression levels of KIFC1 in serous ovarian adenocarcinoma in different tumor grades. **Ci** Kaplan-Meier plots showing overall survival of HGSOC patients based on low or high expression of KIFC1 gene. **Cii** Summary of the number of censored and uncensored values for the Kaplan-Meier survival analysis

Having established a significant correlation between KIFC1 expression and tumor differentiation, we next wanted to determine if there is any association between KIFC1 gene expression and clinical outcomes (overall survival (OS)) for ovarian cancer patients. To this end, we examined single channel microarray data from GEO (GSE9899) [25] to compare the expression levels of KIFC1 among different subtypes. Interestingly, we found that the gene expression levels of KIFC1 were significantly higher in serous ovarian adenocarcinoma (*n* = 154) when compared to all other subtypes (Borderline serous adenocarcinoma, *n* = 18 and Peritoneal serous adenocarcinoma, *n* = 22) (Fig. 2A). Further, we examined grade-wise trends in KIFC1 expression in serous ovarian adenocarcinoma. We observed a significant increase in KIFC1 expression levels with increasing grade (Fig. 2B). OS was calculated as the time interval (in months) from the date of histological diagnosis to date of death from any cause. We then carried out a survival analysis wherein patients were stratified into high- and low-KIFC1 expressing subgroups using the optimal KIFC1 expression cut-point (based on the log-rank test). Irrespective of the histological subtypes (*n* = 284), those with higher KIFC1 expression had shorter OS (*p* < 0.067) than patients with lower KIFC1 (Additional file 1: Figure S2A). To investigate in-depth, we performed a similar survival analysis by stratifying serous ovarian adenocarcinoma patients (*n* = 201) on the basis of site (primary, *n* = 154 and metastatic, *n* = 47) of sample collection. Univariate regression revealed high KIFC1 gene expression correlated significantly (HR = 2.14, *p* = 0.024) with poor OS in primary tumors only (Fig. 2C) but not in metastatic ones (data not shown). This association stayed significant (HR = 2.6, *p* = 0.006) during multivariate analysis when potentially confounding factors like grade and tumor stage were added (Additional file 1: Figure S2B). In sum, enhanced gene expression level of KIFC1 in primary tumors is strongly associated with poor clinical outcomes.

### KIFC1 gene expression correlates with expression of genes related to centrosomal amplification in serous ovarian cancer

Next, we sought to examine the correlation of KIFC1 and genes driving CA. We analyzed expression levels of genes including *CCNA2, CDK1, NEK2, AURKA, MYCN, CCNE2, STIL, LMO4, PLK4, MDM2, CEP63, E2F1, E2F2, E2F3, CEP152, PIM1, PIN1 andCCND1*, whose deregulation is known to drive CA [18, 26–28]. Specifically, we tested the associations between Robust Multi-array Average-normalized expression levels of these genes in primary SOC from 154 patients using Gene Expression Omnibus (GEO) series GSE9899. Higher expression of KIFC1 was significantly correlated with high expression of *CCNA2, CDK1, NEK2, AURKA, E2F2, MYCN, STIL, CCNE2, E2F3, LMO4, PLK4, PIN1 and E2F3* (Table 4). These results suggest that KIFC1 upregulation and enhanced centrosome clustering in the serous ovarian adenocarcinomas may enable tumor cells to manage their increased centrosomal load, avert mitotic catastrophe and promote survival.

**Table 4:** Correlation between expression levels of KIFC1 and genes whose dysregulation drives centrosome amplification

Gene	Pearson correlation	*P*-Value
CCNA2	0.62527	<.0001
NEK2	0.60066	<.0001
E2F1	0.54218	<.0001
CDK1	0.52124	<.0001
E2F2	0.51764	<.0001
AURKA	0.46987	<.0001
STIL	0.397	<.0001
CCNE2	0.36387	<.0001
LMO4	0.36306	<.0001
PLK4	0.34292	<.0001
MYCN	0.31914	<.0001
E2F3	0.31548	<.0001
MDM2	0.24766	0.002
PIN1	0.23016	0.0041
CEP152	0.18128	0.0245
PIM1	0.17826	0.027

**Fig. 3:**
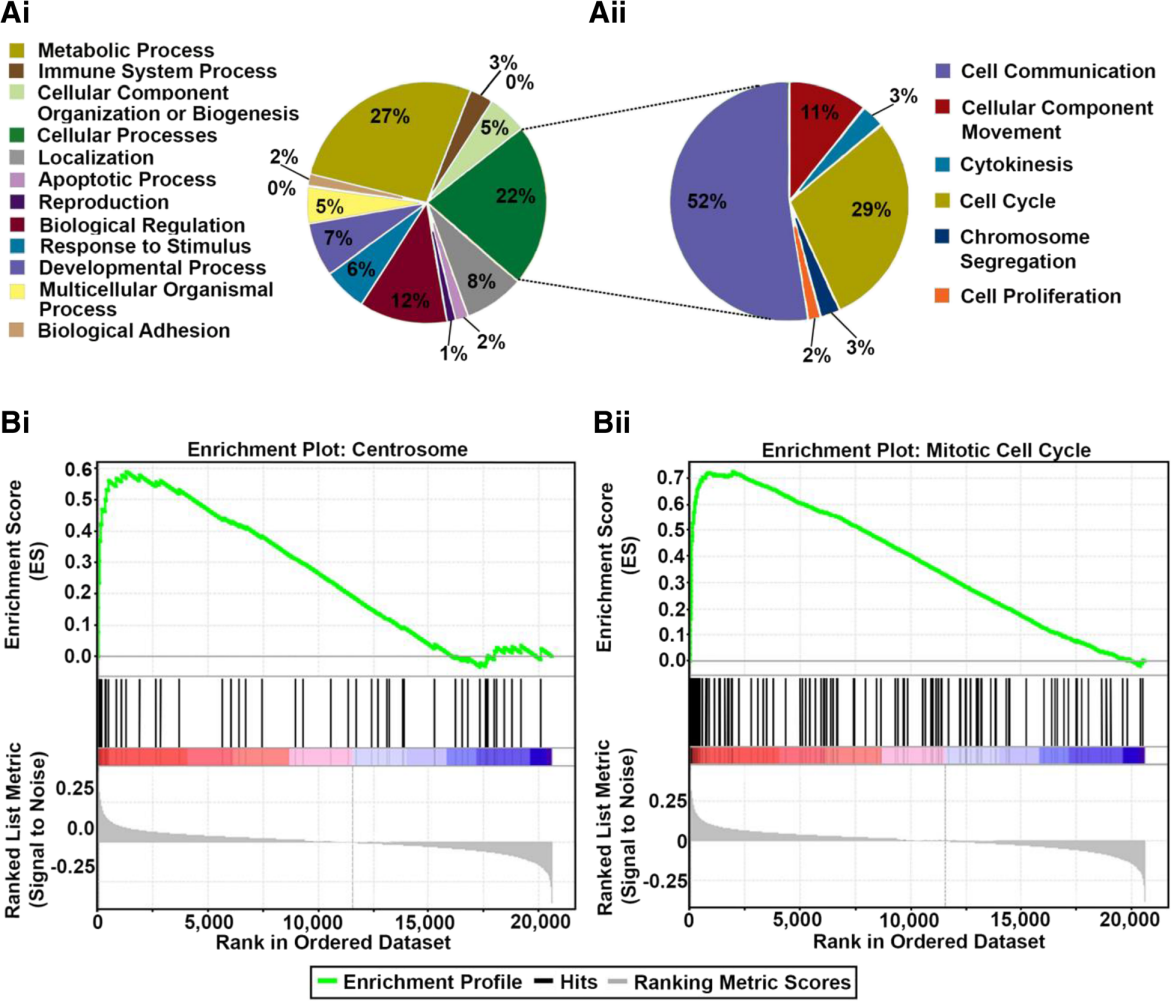
Gene set enrichment analyses for biological processes associated to KIFC1 high group. **Ai** Biological processes enriched in KIFC1 high group. **Aii** Cell cycle processes enriched in KIFC1-high group. **Bi** Enrichment plots of centrosome-related genes. **Bii** Enrichment plot of genes associated with cell cycle progression, with red indicating correlation with the KIFC1-high group and blue the KIFC1-low group

Next, we identified the biological processes which are deregulated in the KIFC1 high risk group. To this end, we probed the publicly-available microarray dataset (GSE9899) and stratified the 154 serous ovarian adenocarcinoma patients from the dataset into KIFC1-high and KIFC1-low groups. We then identified the gene ontologies of significantly overexpressed genes associated with the KIFC1-high group utilizing the PANTHER classification system. When pathway analysis was performed we found that majority of the genes overexpressed were associated to cellular processes like cell communication, cell cycling, cytokinesis and cell proliferation (Fig. 3Ai, ii). We then validated these results by performing the Gene set enrichment analysis (26). We found that KIFC1 high group was significantly (Fdr <0.25 and ES *p* < 0.05) enriched in centrosome and cell cycle gene sets (Fig. 3Bi,ii and Additional file 1: Figure S3A) (see Additional file 1: Tables S1, S2 and S3 for these and all other enriched gene ontologies). The results from GSEA showed that the top 100 gene sets enriched in KIFC1 high group were among the ones which plays key roles in, driving CA (*NEK2, PLK1,CCNA2*), clustering centrosomes (*PRC1*), microtubule spindle (*KIF11, NUSAP1, NUMA1*) etc. Altogether our data shows that the KIFC1-high group had a preponderance of genes representing all four important mitotic kinases –namely Polo-like kinases (PLK1), Aurora kinases (*AURKA, AURK B*), cyclin dependent kinases (*CDK1*) and NIMA related kinases (*NEK1, NEK2*). The coordination of progression through mitosis is mainly orchestrated by protein phosphorylation ensured by these kinases. Thus, it is reasonable to speculate that overexpression of these kinases results in deregulation of the cell cycle resulting in abnormal mitosis that generates cells with aberrant centrosomes and abnormal chromosomal content.

### HGSOC cell lines show higher incidence and severity of centrosome amplification

**Fig. 4:**
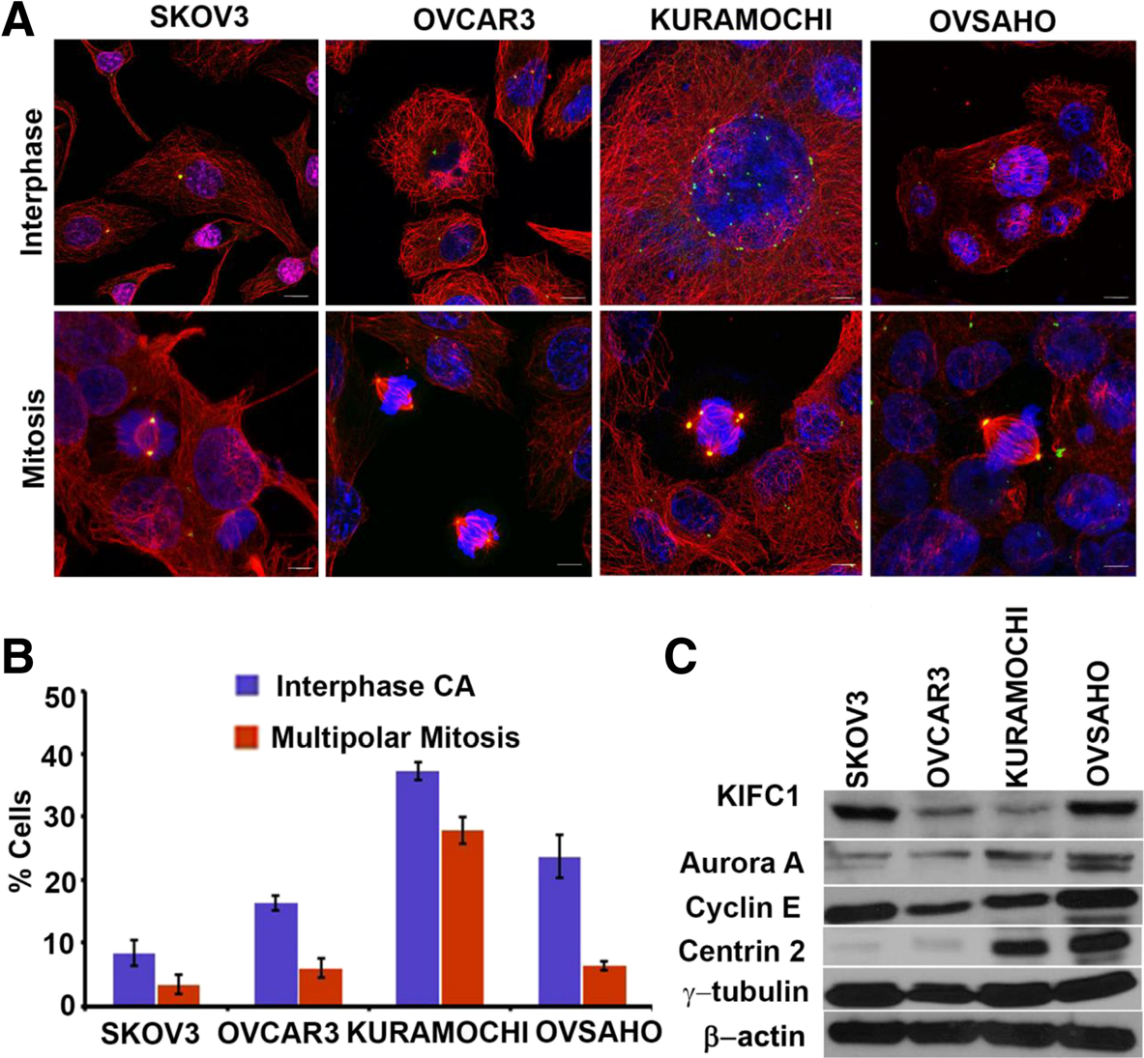
HGSOC cell lines show higher incidence and severity of Centrosome amplification. **a** Confocal microscopic images showing the presence of centrosome amplification and clustering in ovarian cancer cell lines. Centrosomes and microtubules were visualized by immunostaining for γ-tubulin (green) and α-tubulin, respectively, and DNA was stained using DAPI (blue). Scale bar (white) 5 μm. **b** Bar graphical representation of percent cells showing centrosome amplification and multipolar mitosis in human ovarian cancer cell lines. 500 cells were counted in each case. **c** Immunoblots showing the levels of KIFC1 and centrosomal markers in ovarian cancer cells lines (KURAMOCHI, OVCAR3, OVSAHO, and SKOV3)

Having confirmed the association between upregulation of KIFC1 gene and CA genes in HGSOC, we wanted to investigate the CA profile in well-established in vitro cell lines that mimic HGSOC. To this end, we first screened four well-established cancer cell lines (namely, KURAMOCHI, OVCAR3, OVSAHO and SKOV3 by immunostaining centrosomes (γ-tubulin, green) and microtubules (α-tubulin, red) and counterstaining nuclei with DAPI (blue) Fig. 4a. Employing confocal microscopy we imaged 10 areas of interest (at least 500 cells were counted per cell type). Cells with abnormal number (more than two) of gamma tubulin spots were considered as cells with amplified centrosomes. We found that KURAMOCHI exhibited the highest percentage of cells with amplified centrosomes (~38 %) followed by OVSAHO (~24 %), OVCAR3 (~15 %) and SKOV3 (~9 %) (Fig. 4b). In a recent molecular profiling study by Domcke et al., KURAMOCHI and OVSAHO were selected as the representative cell lines for HGSOC [29]. Thus, our findings here parallel previous studies that recognize CA as a biomarker of aggressive tumors. Furthermore, we validated our results by evaluating the expression levels of centrosome-related proteins by performing immunoblotting assays. We found that the cell lines with high CA (KURAMOCHI and OVSAHO) expressed higher levels of centrosome structural proteins (densitometry values for centrin-2 relative to loading control β actin (KURAMOCHI - 0.291445, OVSAHO - 0.432561) and proteins whose dysregulation is known to drive CA (for Cyclin-E and Aurora A, KURAMOCHI- 0.194213 and 0.256828, OVSAHO- 0.428814, 1.664283 respectively) (Fig. 4c). Our next step was to investigate if aberrations in centrosome numbers among the different cell lines had any bearing on the mitotic spindle geometry. Interestingly, we found that the percentage of multipolar mitotic cells in three cell lines (OVSAHO, SKOV3 and OVCAR3) was lower (by ~2 fold) in comparison with the proportion of cells with supernumerary centrosomes (Fig. 4b). This difference in the proportion of cells with CA and multipolar spindles clearly supports the hypothesis that ovarian cancer cells cluster supernumerary centrosomes to form pseudobipolar poles. But as the results here indicate that KURAMOCHI showed significantly more multipolar mitoses when compared to the other ovarian cancer cell lines we tested, we evaluated if there existed variability in the level of clustering molecules that help cancer cells to deal with supernumerary centrosomes by corralling them to form pseudobipolar spindles [24]. To this end, we performed immunoblotting to evaluate expression level of centrosome clustering protein KIFC1 in cell lysates obtained from the ovarian adenocarcinoma cells (KURAMOCHI, OVCAR3, OVSAHO and SKOV3). We found that all the three cell lines with pronounced centrosomal clustering expressed higher levels of KIFC1 (SKOV3- 0.342396, OVCAR3- 0.204796 and OVSAHO- 0.452534) whereas negligible KIFCI expression was noted in KURAMOCHI (0.145452). It is noteworthy to mention that a recent report shows that KURAMOCHI is the only cell line that did not induce tumorigenesis in vivo [11]. This finding resonates with our notion that centrosome clustering is essential for the viability of cancer cells with extra centrosomes and therefore determines their tumorigenicity.

The in vitro findings were validated *in silico* by probing publically-available microarray dataset using Gene set. We interrogated publically-available microarray dataset of ovarian cancer cell lines (GSM133614, GSM133609, GSM887467 and GSM887488). We calculated a cumulative gene expression-based centrosome amplification index (CAI) by adding log-transformed, normalized gene expression for both structural centrosomal proteins (CETN2 (centrin-2), TUBG1 (γ-tubulin), PCNT2 (pericentrin)), and genes implicated in centrosome amplification (PLK4 (polo-like kinase 4) and CCNE1 (cyclin E) genes) (Additional file 1: Fig. S4 Ai). The analysis showed that CAI genes are expressed in all cell lines but is highest in OVSAHO. In addition, we evaluated gene expression levels of KIFC1 and found that the gene expression levels of KIFC1 were higher in cancer cell lines in comparison to normal ovarian surface epithelial cells (Additional file 1: Figure S4 Aii). Taken together, our results indicated that CA and KIFC1 levels are associated with HGSOC cell lines.

## Discussion

Ovarian cancer in the advanced stage remains the deadliest gynecologic malignancy. One of the major causes of the low five-year survival is the diagnosis at later stages after it has already metastasized beyond the pelvis [30]. While extensive literature contains information on the different kinds of biomarkers for ovarian cancer, risk predictive or prognostic markers that are utilized in clinical settings are few and far between. Generally, most researchers focus on single prognostic markers which may be insufficient for complete prognostic information, and also most of them have very low clinical utility. A combination of multiple factors needs to be considered simultaneously to more accurately predict a patient’s prognosis. Presence of heterogeneity in ovarian cancer is another key factor to be considered in prognosis as many ovarian cancer studies have failed to take into account differences in the histological subtype which clearly pose prognostic and therapeutic challenges [30, 31]. Essentially, these unique attributes and challenges can be addressed by personalizing treatments based upon the unique biomarker profiles of individual patients. Thus, a comprehensive understanding of risk predictive or prognostic factors with regard to histological subtype is imperative to devise relevant treatment strategies specific for the particular group of patients or tumor subtypes.

Chromosomal instability (CIN) is the main cause of complex genomic alterations in tumorigenesis. Since CA engenders CIN, the role of CA driven karyotypic diversity is well studied in several malignancies including pancreatic ductal adenocarcinoma, TNBC and colon cancer [18, 20]. Several studies have highlighted the presence of supernumerary centrosomes in ovarian cancer suggesting that CA is a hallmark of ovarian cancer [32–34]. Recently, we also demonstrated the presence of amplified centrosomes in EOC [24]. Supernumerary centrosomes in cancer cells tend to cluster to manage the centrosomal load and thus escape from the perils of mitotic catastrophe. KIFC1 is well studied for its role in clustering supernumerary centrosomes [22, 23]. In our previous study, we emphasized the role of KIFC1 in tumor progression of EOC at the gene expression level [24]. In the present study we have validated those findings by immunostaining ovarian cancer tissue samples for KIFC1. Our findings show that KIFC1 expression increases with the grade in EOC. Among the various subtypes that comprise EOC, we found that KIFC1 expression was highest among high-grade SOC samples. This helped us to focus our study on HGSOC, which is a more prevalent and aggressive form of ovarian cancer. This strong relationship of KIFC1 with HGSOC suggests that KIFC1 may be directly involved in tumor development and in driving aggressiveness by allowing the cancer/poorly differentiated cells to escape mitotic catastrophe and thrive. Moreover, data from our GSEA analysis showed that *BIRC5* gene, which codes for the protein Survivin, that performs dual roles in promoting cell proliferation and preventing apoptosis [35, 36], was among the first 20 enriched genes in KIFC1-high group. Thus, KIFC1 overexpression not only protects cancer cells from undergoing mitotic catastrope but also endows them with low-grade aneuploidy, as a form of genomic instability, and high levels of survival signaling that together facilitate tumor evolution and disease progression. This finding was bolstered by results obtained from *in silico* analysis wherein we found that primary tumors with higher gene expression of KIFC1 were associated with poor survival; by contrast, while samples collected from the metastatic sites showed similar expression levels of KIFC1 as in primary sites, high KIFC1 expression in metastatic sites was not significantly correlated to poor survival. This differential effect of high KIFC1 expression strongly suggests that elevated KIFC1 in primary sites perhaps helps tumor cells present in the primary sites to acquire karyotypic diversity (through CIN), which is more likely to lead to successful metastasis and poor survival. It is possible that once metastasis commences, high KIFC1 levels in the metastatic clones provides little further survival advantage for the cancer cells; alternatively, once metastasis occurs, the survival difference between KIFC1-high and KIFC1-low patients is no longer so marked. Further studies are required to gain more insights into these intriguing issues.

Given the direct association of CA with KIFC1 in the present study, we examined the association of KIFC1 with CA-associated genes. Our in silico analysis indicated that in primary SOC samples KIFC1 expression was positively correlated to the expression of genes which drive CA. CCNA2, NEK2, and AURKA were among the top 10 genes which were highly correlated to KIFC1 expression. Role of NEK2, CCNA2, and AURKA as potential targets in ovarian cancer has been recently highlighted by a detailed systematic bioinformatic study [37]. Besides, this enrichment analysis showed that the KIFC1-high group was enriched in genes implicated in cell cycle regulatory processes, especially genes participating in G2-M transition and the spindle assembly checkpoint (MAD2, BUB1). Several studies in past have reported MAD1 and MAD2 overexpression in different malignancies, and association of this overexpression with aneuploidy and poor overall survival [38–40]. Thus, our findings from the GSEA and Pathway analysis suggests that KIFC1 overexpression drives overexpression of genes that control mitotic checkpoints (Additional file 1: Table S2), which by generating aneuploidy, accelerate tumor progression and evolution of more aggressive phenotypes.

In line with these *in silico* findings, we found that cell line derived from HGSOC displayed robust CA, and the proteins which are known to drive CA were also highly expressed. Some recent studies on molecular profiling of ovarian cell lines have demonstrated that OVSAHO represents most of the characteristics (*KRAS, p53* and *BRCA1* and 2 mutations) of HGSOC [29] and is considered to be most aggressive cell line among all. From our study, we found that OVSAHO cells expressed the highest levels of KIFC1 and in spite of presence of interphase supernumerary centrosomes it showed significantly low level of multipolar mitosis. These findings clearly indicate that strong association of CA and clustering with KIFC1 overexpression, which leads to CIN, could be the underlying cause of aggressiveness in these cells. Testing effects of centrosome declustering drugs on these cells could prove to be advantageous.

## Conclusions

Taken together our results indicate that HGSOC overexpresses KIFC1, which is associated with poor overall survival suggesting a causative link between KIFC1 and tumor aggressiveness. These findings highlight KIFC1 as a potential biomarker to predict disease aggressiveness KIFC1 may also serve as a cancer-selective therapeutic target for high-grade serous ovarian adenocarcinoma patients.

## Methods

### Cell culture

The four ovarian cancer cell lines primarily utilized in this study included OVCAR3, KURAMOCHI, SKOV3, and OVSAHO. The SKOV3 and OVCAR3 cell lines were obtained from ATCC and KURAMOCHI, and OVSAHO were obtained from JCRB. All the cell lines were cultured according to the instructions given by the company.

### Immunohistochemistry and scoring

Formalin-fixed paraffin-embedded tissue microarrays (TMAs) for ovarian cancer were obtained from US, Biomax, Inc. Company provided the ethical statement to confirm that, all the participants provided their written consents and patient privacy and anonymity was maintained. TMAs were deparaffinized in a 60 °C oven for 20 min and placed in 3 consecutive xylene washes. Rehydration of the slides were carried out by putting them through a series of washes involving different concentrations of ethanol in water - 100 %, 95 %, 70 %, and 50 % - for 3 min each. The antigen retrieval process was done using a pressure cooker and 0.01 M citrate buffer with a pH of 6.1. The slides were heated at a temperature of 120 °C for 30 min. After cooling in ice for 20 min, the slides were first subjected to hydrogen peroxide blocking and then protein blocking (both obtained from ThermoScientific) for 20 min and 10 min, respectively. Tissues were incubated with anti-KIFC1 antibody (Abcam) for 1 h, before incubating with MACH2 HRP-conjugated secondary antibody (Biocare Medical) for 30 min. Enzymatic antibody detection using Betazoid DAB Chromogen Kit (Biocare Medical) was followed by nuclear staining with Myer’s hematoxylin (Dako). The staining intensity was scored as 0 = none, 1 = low, 2 = moderate, or 3 = high, and the percentage of KifC1-positive cells from 10 randomly selected fields (~500 cells) was determined. The product of the staining intensity and the percent of positive cells constituted the WI. Statistical analysis was performed using – Tukey’s post hoc test.

### Cell staining and imaging

Cells were cultured on coverslips and, after the confluency reached approximately 80 %, the cells were fixed with ice-cold methanol for 7 min. The cells were blocked with 5 % BSA/0.01 % Triton X for 45 min at room temperature and then incubated at 37 °C with antibodies directed against γ-tubulin and α-tubulin at a dilution of 1:2000 for 30 min. The cells underwent quick washes 5 times with 1xPBS before being incubated with Alexa Fluor 488 anti-mouse and Alexa Fluor 555 anti-rabbit at a dilution of 1:2000 at 37 °C for 30 min. After washing the cells 8 times with 1x PBS briefly, the cells were then incubated with Hoechst 33342 (1:5000 dilution) at room temperature for 10 min. The cells were mounted with Prolong-Gold antifade reagent after being washed with 1x PBS 3 times and observed using Zeiss LSM 700 Confocal microscope (Oberkochen, Germany) and the images were processed with ZEN software (Oberkochen, Germany).

### Immunoblotting

Cell lysates were prepared from 80 % confluent cells by scraping with 250ul of 1x lysis prepared from 10x cell lysis buffer (Cell Signaling). The 1x lysis buffer contained 1 mM b-glycerophosphate, 20 mM Tris–HCl (pH 7.5), 1 mM Na2EDTA, 1 mM Na3VO4, 150 mM NaCl, 1 mM EGTA, 2.5 mM Na4P2O7, 1ug/ml leupeptin, and 1 % Triton. Cell lysates were fractionated using 10 % SDS-PAGE gel. The samples were allowed to run at 70 V for 90 min. Protein transfer onto polyvinylidene difluoride (PVDF) membrane was done for 2 h via the wet transfer method at 70 V. The membrane was then blocked in 5 % non-fat, dry milk in 1x TBST for 1 h at room temperature and probed with the relevant antibodies at a dilution of 1:1000 overnight at 4 °C. Primary antibody incubation was followed by incubation with the corresponding secondary antibody at a dilution of 1: 10,000 for 1 h at room temperature. SuperSignal West Pico Chemiluminescent Substrate (ThermoScientific) was directly applied to the membrane for the subsequent analysis. Cyclin E and Centrin-2 antobodies were obtained from Santa Cruz Biotech, γ-tubulin from Dako, and KIFC1 and Aurora A antibodies from Abcam.

### *In silico* analysis

One channel microarray data was downloaded from gene expression omnibus (GEO) database for primary ovarian cancer samples GSE 9899 [25]. Data was Mas5.0 normalized and was further taken for processing. Logarithm to the base 2-transformed KIFC1 expression levels from all ovarian cancer samples (*n* = 284) regardless of histotypes were extracted from GEO database. Further analysis were carried only on the serous adenocarcinoma samples (*n* = 200). Overall survival (OS) was calculated as the time interval (in months) from the date of histological diagnosis to date of death from any cause. KIFC1 was categorized into high and low groups based on the optimal overall survival cut - points using the log-rank test.

### Public microarray data analysis

Robust Multi-array Average normalized expression levels of KIFC1 and genes which drive CA (*CCNA2, CDK1, NEK2, AURKA, MYCN*, *CCNE2*, *STIL, LMO4, PLK4, MDM2, CEP63, E2F1, E2F2, E2F3, CEP152, PIM1, PIN1, CCND1)* from the primary serous ovarian carcinoma of 154 patients were obtained from GEO series GSE 9899 To obtain Pearson’s correlation coefficients between genes whose dysregulation drives CA, SAS software (IBM) was used for the analyses, with *p* < 0.05 indicating statistical significance.

### Gene set enrichment analysis of public microarray data

Publicly available pre-processed gene expression profiles of primary ovarian tumors (*n* = 154 from Tothill dataset [25], GSE9899; Patients were stratified into two groups by KIFC1 score. Patients with KIFC1 expressions below the optimal KIFC1 survival threshold where placed in the low-risk group whereas the above threshold patients where stratified to the high-risk group. GSEA was performed as indicated in studies by Tamayo, et al. (2005, PNAS 102, 15545–15550) and Mootha, Lindgren, et al. (2003, Nat Genet 34, 267–273). False discovery rate q-values.25 were considered statistically significant.

### In silico analysis of KIFC1 gene expression and centrosomal amplification index (CAI) genes in cell lines

One channel microarray data was downloaded from GEO database for four cell lines with GSM ids GSM133614, GSM133609, GSM887467 and GSM887488 namely, Ovcar-5, SKOV3, OVSAHO, and OVCAR3 respectively. Data was Mas5.0 normalized and was further taken for processing. Logarithm to the base 2 transformed KIFC1 and expression levels from ovarian cell lines were extracted from the GEO database. PLK4, Aur-A, Aur B, Cyclin E, Centrin, γ-tubulin and pericentrin genes expression values were added to make centrosomal amplification index.

### Statistical analysis

Statistical analyses were performed using two-tailed Student’s t-tests, Anova and Tukey’s post hoc tests. The criterion for statistical significance for all analyses was *p* < 0.05. Standard errors were calculated using the general Excel formula where we divided the standard deviation by the square root of the number of samples. Kaplan-Meier analysis and Cox regression were performed using SPSS (IBM). Optimal cut-points were identified with the stratification which gave the largest log-rank *χ* ^2^ value.

## Additional file

Additional file 1: Supplementary Figures and Tables (DOCX 811 kb)

## Abbreviations

CA: Centrosome amplification
CAI: centrosome amplification Index
CIN: chromosomal instability
EOC: epithelial ovarian cancer
GSEA: gene set enrichment analysis
GEO: gene expression omnibus
HGSOC: High grade serous ovarian adenocarcinoma
OS: overall survival
SOC: serous ovarian adenocarcinoma
TCGA: the cancer genome atlas
TNBC: Triple negative breast cancer
WI: Weighted index

## References

[1] Siegel R, Ma J, Zou Z (2014). Cancer statistics, 2014. CA Cancer J Clin.

[2] Bast RC, Hennessy B, Mills GB (2009). The biology of ovarian cancer: new opportunities for translation. Nat Rev Cancer.

[3] Schmid BC, Oehler MK (2014). New perspectives in ovarian cancer treatment. Maturitas.

[4] Yap TA, Carden CP, Kaye SB (2009). Beyond chemotherapy: targeted therapies in ovarian cancer. Nat Rev Cancer.

[5] Vaughan S, Coward JI, Bast RC (2011). Rethinking ovarian cancer: recommendations for improving outcomes. Nat Rev Cancer.

[6] Auersperg N, Edelson MI, Mok SC (1998). The biology of ovarian cancer. Semin Oncol.

[7] Auersperg N (2011). The origin of ovarian carcinomas: a unifying hypothesis. Int J Gynecol Pathol.

[8] Bell D, Berchuck A, Birrer M, Chien J, Cramer D, Dao F, et al. Integrated genomic analyses of ovarian carcinoma. Nature. 2011;474: 609–615. 10.1038/nature10166 PMC3163504 21720365

[9] Koboldt DC, Fulton RS, McLellan MD, Schmidt H, KalickiVeizer J, McMichael JF, et al. Comprehensive molecular portraits of human breast tumours. Nature. 2012;490: 61–70. 10.1038/nature11412 PMC3465532 23000897

[10] Fleury H, Communal L, Carmona E (2015). Novel high-grade serous epithelial ovarian cancer cell lines that reflect the molecular diversity of both the sporadic and hereditary disease. Genes Cancer.

[11] Mitra AK, Davis DA, Tomar S (2015). In vivo tumor growth of high-grade serous ovarian cancer cell lines. Gynecol Oncol.

[12] Scully R (2000). Role of BRCA gene dysfunction in breast and ovarian cancer predisposition. Breast Cancer Res.

[13] Liu Y, Kulesz-Martin M (2001). p53 protein at the hub of cellular DNA damage response pathways through sequence-specific and non-sequence-specific DNA binding. Carcinogenesis.

[14] Venkitaraman AR (2002). Cancer susceptibility and the functions of BRCA1 and BRCA2. Cell.

[15] Pils D, Bachmayr-Heyda A, Auer K (2014). Cyclin E1 (CCNE1) as independent positive prognostic factor in advanced stage serous ovarian cancer patients - a study of the OVCAD consortium. Eur J Cancer.

[16] Lassus H, Staff S, Leminen A (2011). Aurora-A overexpression and aneuploidy predict poor outcome in serous ovarian carcinoma. Gynecol Oncol.

[17] Landen CN, Lin YG, Immaneni A (2007). Overexpression of the centrosomal protein Aurora-A kinase is associated with poor prognosis in epithelial ovarian cancer patients. Clin Cancer Res.

[18] Mittal K, Ogden A, Reid MD (2015). Amplified centrosomes may underlie aggressive disease course in pancreatic ductal adenocarcinoma. Cell Cycle.

[19] Godinho SA, Picone R, Burute M (2014). Oncogene-like induction of cellular invasion from centrosome amplification. Nature.

[20] Pannu V, Mittal K, Cantuaria G (2015). Rampant centrosome amplification underlies more aggressive disease course of triple negative breast cancers. Oncotarget.

[21] Basto R, Brunk K, Vinadogrova T (2008). Centrosome amplification can initiate tumorigenesis in flies. Cell.

[22] Pannu V, Rida PC, Ogden A (2015). HSET overexpression fuels tumor progression via centrosome clustering-independent mechanisms in breast cancer patients. Oncotarget.

[23] Li Y, Lu W, Chen D (2015). KIFC1 is a novel potential therapeutic target for breast cancer. Cancer Biol Ther.

[24] Pawar S, Donthamsetty S, Pannu V (2014). KIFCI, a novel putative prognostic biomarker for ovarian adenocarcinomas: delineating protein interaction networks and signaling circuitries. J Ovarian Res.

[25] Tothill RW, Tinker AV, George J (2008). Novel molecular subtypes of serous and endometrioid ovarian cancer linked to clinical outcome. Clin Cancer Res.

[26] Leontovich AA, Salisbury JL, Veroux M (2013). Inhibition of Cdk2 activity decreases Aurora-A kinase centrosomal localization and prevents centrosome amplification in breast cancer cells. Oncol Rep.

[27] Montanez-Wiscovich ME, Shelton MD, Seachrist DD (2010). Aberrant expression of LMO4 induces centrosome amplification and mitotic spindle abnormalities in breast cancer cells. J Pathol.

[28] Marina M, Saavedra HI (2014). Nek2 and Plk4: prognostic markers, drivers of breast tumorigenesis and drug resistance. Front Biosci (Landmark Ed).

[29] Domcke S, Sinha R, Levine DA (2013). Evaluating cell lines as tumour models by comparison of genomic profiles. Nat Commun.

[30] Agarwal R, Kaye SB (2005). Prognostic factors in ovarian cancer: how close are we to a complete picture?. Ann Oncol.

[31] Davidson B, Trope CG (2014). Ovarian cancer: diagnostic, biological and prognostic aspects. Womens Health (Lond Engl).

[32] Hsu LC, Kapali M, DeLoia JA (2005). Centrosome abnormalities in ovarian cancer. Int J Cancer.

[33] Bayani J, Paderova J, Murphy J (2008). Distinct patterns of structural and numerical chromosomal instability characterize sporadic ovarian cancer. Neoplasia.

[34] Zhang Y, Tian Y, Yu JJ (2013). Overexpression of WDR62 is associated with centrosome amplification in human ovarian cancer. J Ovarian Res.

[35] Or YY, Chow AK, Ng L (2014). Survivin depletion inhibits tumor growth and enhances chemosensitivity in hepatocellular carcinoma. Mol Med Rep.

[36] Plewka D, Jakubiec-Bartnik B, Morek M (2015). Survivin in ovary tumors. Ginekol Pol.

[37] Ye Q, Lei L, Aili AX (2015). Identification of potential targets for ovarian cancer treatment by systematic bioinformatics analysis. Eur J Gynaecol Oncol.

[38] Alizadeh AA, Eisen MB, Davis RE (2000). Distinct types of diffuse large B-cell lymphoma identified by gene expression profiling. Nature.

[39] Baker DJ, Jeganathan KB, Cameron JD (2004). BubR1 insufficiency causes early onset of aging-associated phenotypes and infertility in mice. Nat Genet.

[40] Bharadwaj R, Yu H (2004). The spindle checkpoint, aneuploidy, and cancer. Oncogene.

