# Supplementary material for: A centrosome clustering protein, KIFC1, predicts aggressive disease course in serous ovarian adenocarcinomas
Source: J Ovarian Res. 2016 Mar 18;9:17. doi: 10.1186/s13048-016-0224-0 (PMC5477851; doi:10.1186/s13048-016-0224-0)
Supplement: Additional file 1: — Supplementary Figures and Tables (DOCX 811 kb) [file 13048_2016_224_MOESM1_ESM.docx]

**Supplementary Figures and Tables**

**Figure S1**


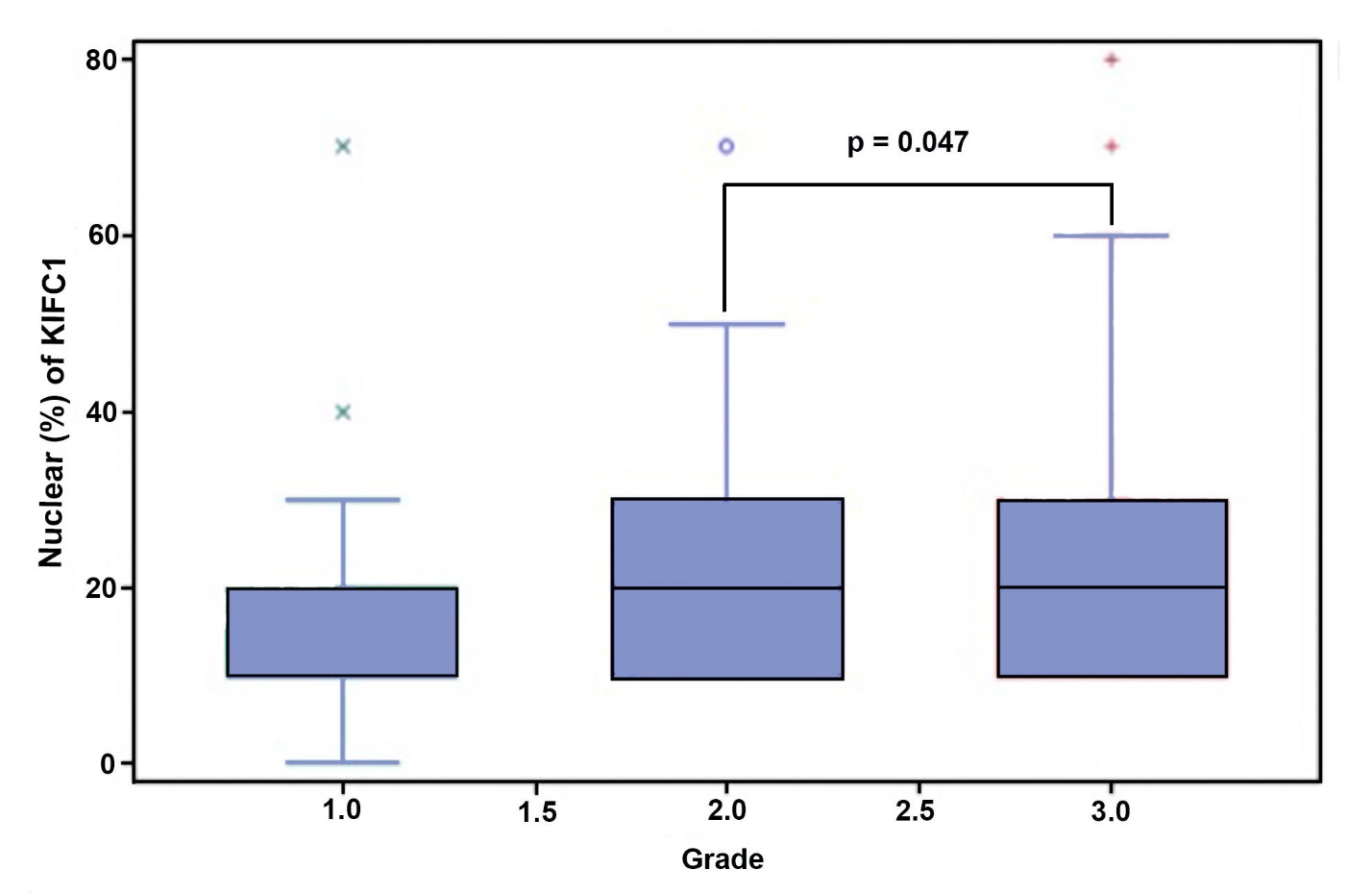


**Figure S1:** High grade serous ovarian carcinomas exhibit higher expression of KIFC1**. A.** Box-whisker plot representing the weighted index for KIFC1 expression in low and high-grade SOC tissues.

**Figure S2A**


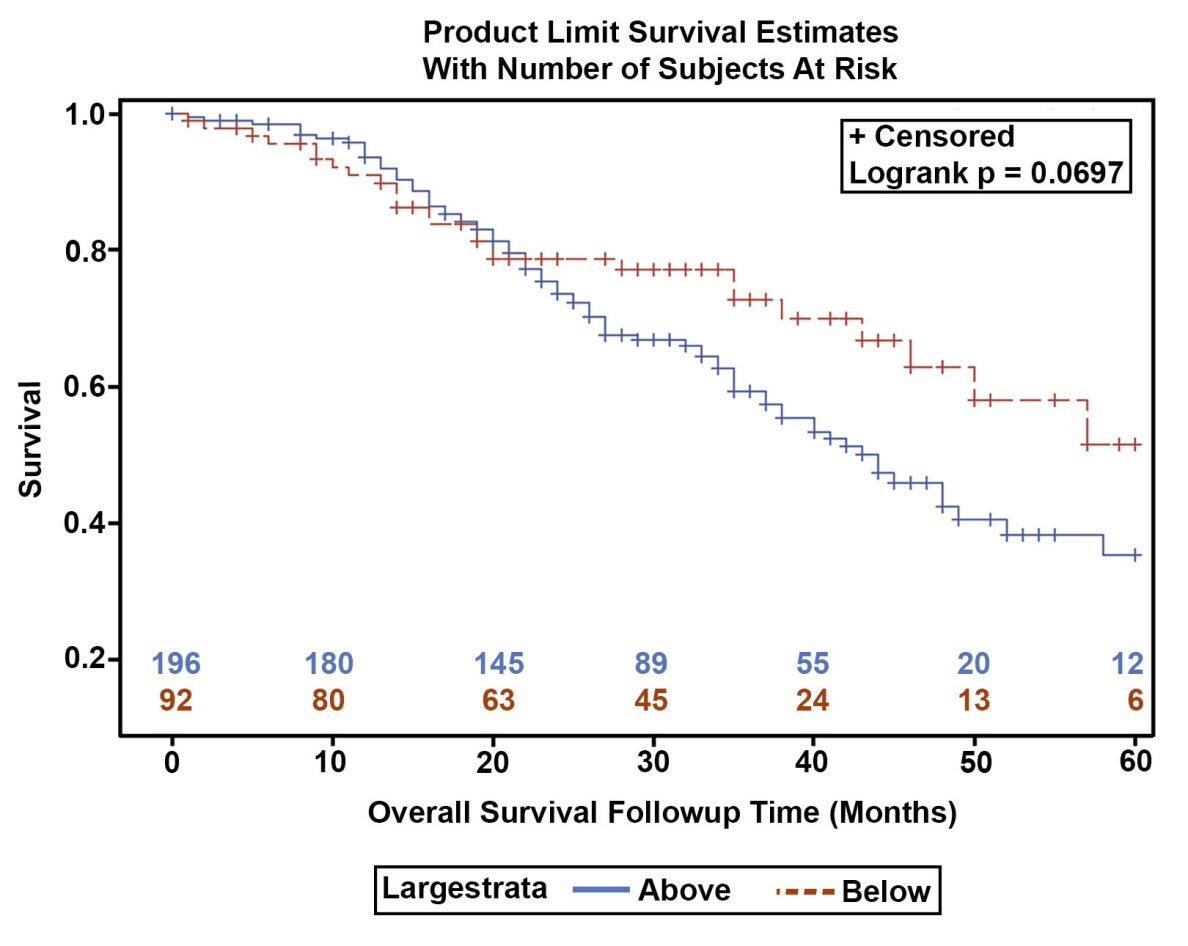


**Figure. S2A** KIFC1 expression is associated with poor overall survival **A**. Kaplan-Meier plots of overall survival based on low or high expression of KIFC1 gene in ovarian cancer patients regardless of histotypes.

**Figure S2B**

**
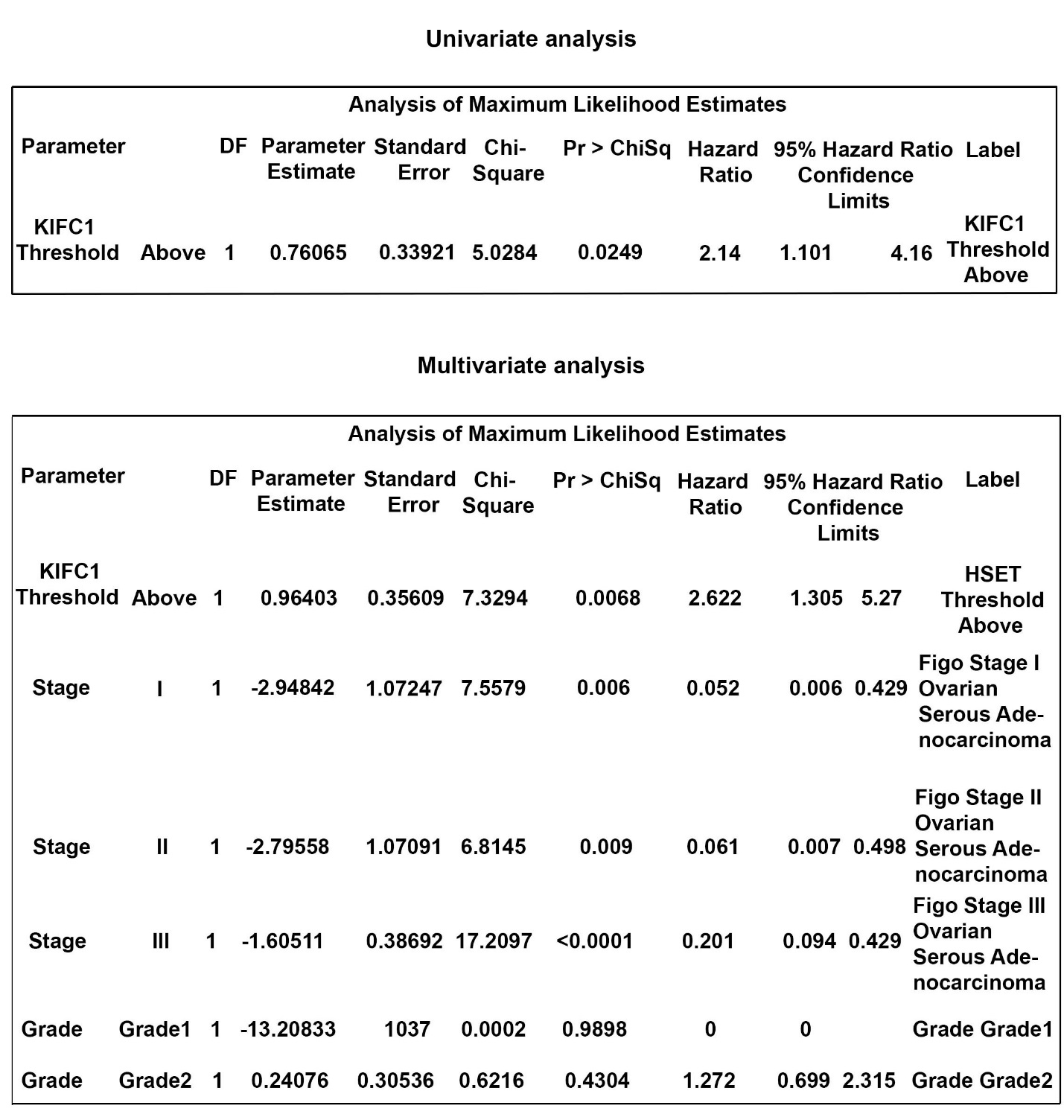
**

**Figure S2B:** Descriptive table for univariate and multivariate analysis.

**Figure S3**


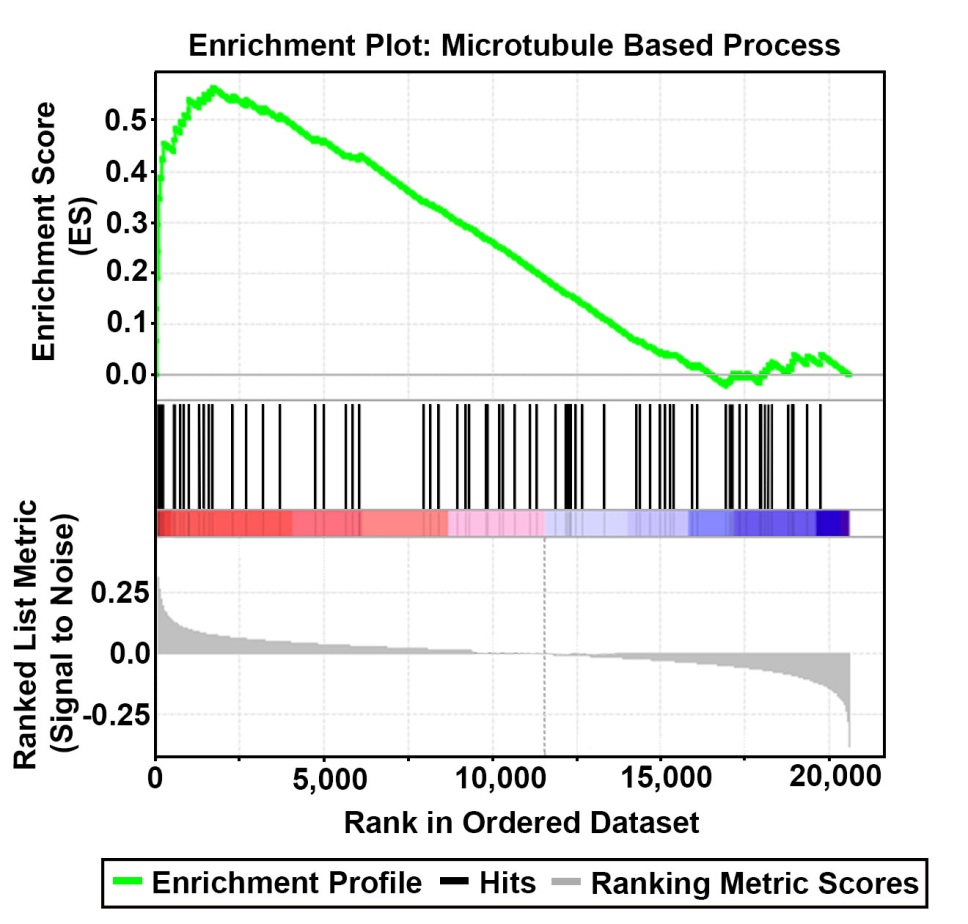


**Figure S3.** Gene set enrichment analyses for biological processes associated to KIFC1 high group. **A.** Enrichment plot of genes associated to microtubule based processes, with red indicating correlation with the KIFC1-high group and blue the KIFC1-low group.

**Figure S4**


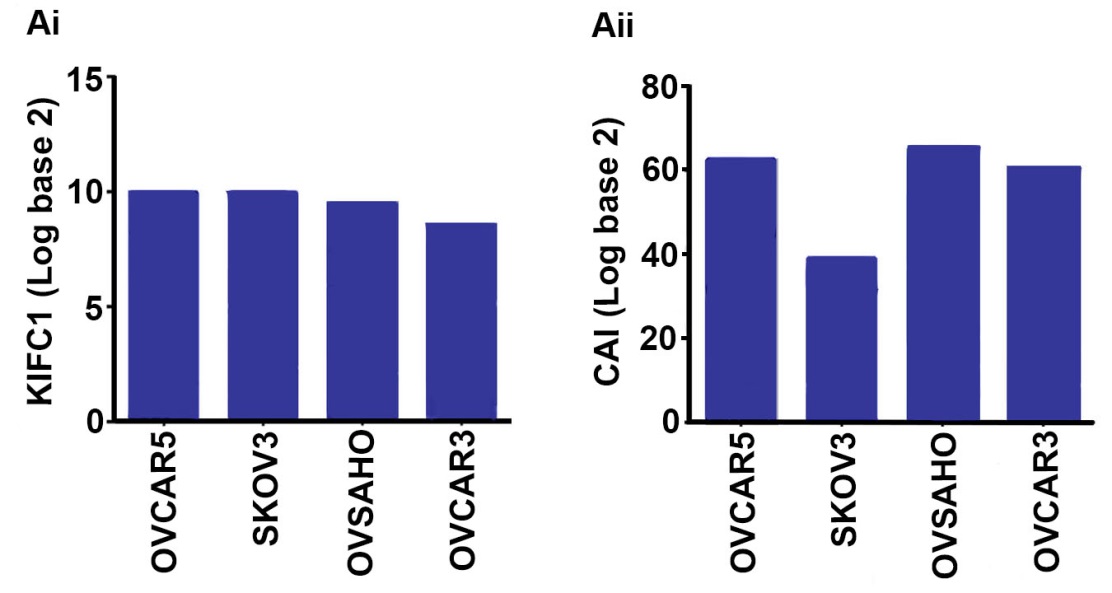


**Figure S4. HGSOC cell lines show higher expression of genes, driving Centrosome amplification, and KIFC1 *in silico*. Ai.** Bar graph representing Log Base 2 KIFC1expression in ovarian cell lines (Ovcar5, OVCAR3, OVSAHO, and SKOV3). **Aii** Bar graph representing Log Base 2 Centrosome amplification index expression in ovarian cell lines (OVCAR5, OVCAR3, OVSAHO, and SKOV3).

**Table S1**

| **GENE SYMBOL** | **RANK IN GENE LIST** | **RANK METRIC SCORE** | **RUNNING ES** | **CORE ENRICHMENT** |
| --- | --- | --- | --- | --- |
|  |  |  |  |  |
| TOP2A | 25 | 0.334119827 | 0.0778286 | Yes |
| KIF15 | 31 | 0.329936087 | 0.15564038 | Yes |
| BIRC5 | 33 | 0.328538954 | 0.23331621 | Yes |
| NEK2 | 49 | 0.306233615 | 0.30503407 | Yes |
| PLK1 | 69 | 0.277375519 | 0.3697302 | Yes |
| CTAG2 | 133 | 0.232606009 | 0.42169446 | Yes |
| ESPL1 | 163 | 0.211262807 | 0.47026342 | Yes |
| SAC3D1 | 317 | 0.161001891 | 0.50090957 | Yes |
| NDE1 | 394 | 0.147750571 | 0.5321666 | Yes |
| BRCA2 | 470 | 0.137022048 | 0.5609342 | Yes |
| CROCC | 841 | 0.106630601 | 0.56816083 | Yes |
| AZI1 | 1055 | 0.095914356 | 0.58048993 | Yes |
| CKAP5 | 1293 | 0.087143637 | 0.58957654 | Yes |

**Table S1**. Rank-ordered list of filtered genes comprising centrosome components with associated rank metric scores, enrichment scores (ES), and whether each gene is part of the core enriched genes (i.e., the leading-edge subset) in the KIFC1-high group.

**Table S2**

| **GENE SYMBOL** | **RANK IN GENE LIST** | **RANK METRIC SCORE** | **RUNNING ES** | **CORE ENRICHMENT** |
| --- | --- | --- | --- | --- |
|  |  |  |  |  |
| KIF23 | 7 | 0.380206674 | 0.026178278 | Yes |
| KIF2C | 16 | 0.34368071 | 0.04975993 | Yes |
| NCAPH | 19 | 0.341490924 | 0.07348197 | Yes |
| KIF15 | 31 | 0.329936087 | 0.095958345 | Yes |
| BIRC5 | 33 | 0.328538954 | 0.118825816 | Yes |
| TPX2 | 43 | 0.312425733 | 0.14017852 | Yes |
| BUB1 | 45 | 0.311746061 | 0.16187464 | Yes |
| ANLN | 47 | 0.309315741 | 0.18340124 | Yes |
| PRC1 | 48 | 0.307584703 | 0.20485595 | Yes |
| NEK2 | 49 | 0.306233615 | 0.22621644 | Yes |
| CDKN3 | 52 | 0.303843647 | 0.24731249 | Yes |
| CDC6 | 54 | 0.301827401 | 0.26831678 | Yes |
| UBE2C | 58 | 0.294732958 | 0.28872848 | Yes |
| TTK | 59 | 0.294727236 | 0.30928636 | Yes |
| NUSAP1 | 65 | 0.281409442 | 0.328671 | Yes |
| CENPF | 68 | 0.277548671 | 0.34793293 | Yes |
| PLK1 | 69 | 0.277375519 | 0.3672805 | Yes |
| CDC7 | 72 | 0.27584514 | 0.3864236 | Yes |
| CENPE | 76 | 0.275140435 | 0.4054687 | Yes |
| AURKA | 77 | 0.274723768 | 0.4246313 | Yes |
| FBXO5 | 86 | 0.270314395 | 0.44309548 | Yes |
| CDC25C | 93 | 0.264975429 | 0.46128497 | Yes |
| BUB1B | 106 | 0.255380183 | 0.47851205 | Yes |
| KIF11 | 126 | 0.236126006 | 0.49405414 | Yes |
| CCNA2 | 128 | 0.235330373 | 0.5104201 | Yes |
| CDCA5 | 131 | 0.234785706 | 0.5266992 | Yes |
| RCC1 | 157 | 0.2154641 | 0.54050696 | Yes |
| ESPL1 | 163 | 0.211262807 | 0.55499876 | Yes |
| ZWINT | 185 | 0.202192873 | 0.5680762 | Yes |
| CDK2 | 201 | 0.19581379 | 0.5810018 | Yes |
| CDKN2A | 207 | 0.191838071 | 0.5941387 | Yes |
| STMN1 | 238 | 0.180245936 | 0.6052456 | Yes |
| CIT | 255 | 0.175560325 | 0.61670965 | Yes |
| KNTC1 | 264 | 0.173231944 | 0.6284021 | Yes |
| MAD2L1 | 305 | 0.16407761 | 0.6378927 | Yes |
| CDKN2D | 327 | 0.159156412 | 0.6479683 | Yes |
| KIF22 | 343 | 0.157049492 | 0.65819 | Yes |
| E2F1 | 390 | 0.147849038 | 0.66625553 | Yes |
| SKP2 | 420 | 0.144821107 | 0.67494035 | Yes |
| MAD2L2 | 450 | 0.140390754 | 0.6833162 | Yes |
| DBF4 | 488 | 0.135220751 | 0.6909405 | Yes |
| CDKN2C | 553 | 0.128658727 | 0.696788 | Yes |
| POLD1 | 564 | 0.127196997 | 0.70517176 | Yes |
| POLE | 710 | 0.11385873 | 0.7060298 | Yes |
| DDX11 | 726 | 0.113228329 | 0.7131949 | Yes |
| PKMYT1 | 774 | 0.109883375 | 0.7185633 | Yes |
| SMC4 | 852 | 0.105742373 | 0.7221773 | Yes |
| PIN1 | 1119 | 0.093436472 | 0.71569943 | Yes |
| TBRG4 | 1304 | 0.086835429 | 0.71276724 | Yes |
| ASNS | 1397 | 0.084191032 | 0.7141451 | Yes |
| SMC1A | 1575 | 0.079489306 | 0.71104246 | Yes |
| PPP5C | 1741 | 0.076085858 | 0.70828867 | Yes |
| CDC25B | 1758 | 0.075692296 | 0.7127867 | Yes |
| CDC27 | 1859 | 0.073668718 | 0.7130398 | Yes |
| POLA1 | 1913 | 0.072428539 | 0.71550256 | Yes |
| BTG3 | 1918 | 0.072311886 | 0.72035104 | Yes |
| GSPT1 | 1927 | 0.072142698 | 0.72499233 | Yes |

**Table S2**. Rank-ordered list of filtered genes associated to mitotic cycle components with associated rank metric scores, enrichment scores (ES), and whether each gene is part of the core enriched genes (i.e., the leading-edge subset) in the KIFC1-high group.

**Table S3**

| **GENE SYMBOL** | **RANK IN GENE LIST** | **RANK METRIC SCORE** | **RUNNING ES** | **CORE ENRICHMENT** |
| --- | --- | --- | --- | --- |
|  |  |  |  |  |
| KIF23 | 7 | 0.380206674 | 0.068447836 | Yes |
| KIF2C | 16 | 0.34368071 | 0.13023852 | Yes |
| KIF4A | 32 | 0.329859108 | 0.18918754 | Yes |
| PRC1 | 48 | 0.307584703 | 0.24410658 | Yes |
| TTK | 59 | 0.294727236 | 0.29694292 | Yes |
| NUSAP1 | 65 | 0.281409442 | 0.34761333 | Yes |
| KIF11 | 126 | 0.236126006 | 0.38741168 | Yes |
| RCC1 | 157 | 0.2154641 | 0.42493314 | Yes |
| STMN1 | 238 | 0.180245936 | 0.45364717 | Yes |
| TUBG1 | 542 | 0.129438803 | 0.46230626 | Yes |
| KIF1A | 577 | 0.125762284 | 0.4834036 | Yes |
| KIF1B | 729 | 0.112902582 | 0.496475 | Yes |
| NLGN1 | 846 | 0.106165938 | 0.51003253 | Yes |
| YKT6 | 972 | 0.098996036 | 0.5218544 | Yes |
| MAP1S | 976 | 0.098900363 | 0.5396018 | Yes |
| CKAP5 | 1293 | 0.087143637 | 0.5399754 | Yes |
| SNAP29 | 1408 | 0.083875522 | 0.54959744 | Yes |
| SMC1A | 1575 | 0.079489306 | 0.5558929 | Yes |
| CENPJ | 1691 | 0.076832272 | 0.56419194 | Yes |

**Table S3**. Rank-ordered list of filtered genes associated microtubule based processes with associated rank metric scores, enrichment scores (ES), and whether each gene is part of the core enriched genes (i.e., the leading-edge subset) in the KIFC1-high group.
